# The Movement Profile of Habitual Vacuuming as a Cyclic Movement—A Pilot Study

**DOI:** 10.3390/ijerph17238793

**Published:** 2020-11-26

**Authors:** Christian Maurer-Grubinger, Jasmin Haenel, Laura Fraeulin, Fabian Holzgreve, Eileen M. Wanke, David A. Groneberg, Daniela Ohlendorf

**Affiliations:** Institute of Occupational Medicine, Social Medicine and Environmental Medicine, Goethe University Frankfurt, 60590 Frankfurt, Germany; christian.maurer.cm@gmail.com (C.M.-G.); j.lampe@med.uni-frankfurt.de (J.H.); fraeulin@med.uni-frankfurt.de (L.F.); wanke@med.uni-frankfurt.de (E.M.W.); occup-med@uni-frankfurt.de (D.A.G.); Ohlendorf@med.uni-frankfurt.de (D.O.)

**Keywords:** vacuuming, cleaning, movement profile, movement pattern, inertial motion capture, musculoskeletal disorders, ergonomics

## Abstract

Background: Vacuum cleaning, which is associated with musculoskeletal complaints, is frequently carried out in private households and by professional cleaners. The aim of this pilot study was to quantify the movements during habitual vacuuming and to characterize the movement profile with regard to its variability. Methods: The data were collected from 31 subjects (21 f/10 m) using a 3D motion analysis system (XSens). Eight vacuum cleaners were used to vacuum polyvinyl chloride (PVC) and carpet floors. In 15 joints of the right upper extremity, the trunk and the lower extremities, Principal Component Analysis was used to determine the predominantly varying joints during vacuuming. Results: The movements of the trunk and the lower extremities were relatively constant and, therefore, had less influence. The shoulder, elbow and wrist joints were identified as joints that can be decisive for the movement profile and that can be influenced. These joints were represented in the course of the vacuuming cycle by the mean movement with its standard deviation. Conclusion: In summary, the generalization of a movement profile is possible for the trunk and the lower extremities due to the relative homogeneity. In future it will be necessary to identify factors influencing variability in order to draw conclusions about movement ergonomics.

## 1. Introduction

Household cleaning tasks are part of regular, everyday activities that are carried out frequently. In Germany people spend an average of 2.25 h a week cleaning their household [1] and 49% of Germans have their household vacuumed several times a week [2]. According to an international survey, 49% of respondents vacuum at least twice a week [3]. The duration of vacuuming was found to be less than 30 min for 43% and from 30 min up to one hour for 46% [3]. In a survey of cleaners in the UK, 94% reported using a vacuum cleaner every day, with an average duration of 82.5 ± 38.9 min [4].

The risks from biomechanical load on the upper extremities in household tasks are assessed as light to moderate [5]. However, if activities are frequently repeated, this can lead to musculoskeletal disorders in the long term [6]. Lim et al. [7] reported that pain occurred during vacuuming in 77.4% (*n* = 119) of women interviewed who frequently used vacuum cleaners, 80% of whom had pain in their arms and shoulders. In addition, vacuuming was, for 12.3%, one of the most frequently reported tasks of an online survey on household tasks that led to fatigue [8]. On a scale from 1 (“not tiring”) to 4 (“extremely tiring”), the degree of perceived fatigue was rated as “slightly tiring” with an average of 2 [8].

Musculoskeletal complaints that can be caused by cleaning tasks lead to work-related complaints, especially for professional cleaners. The prevalence of musculoskeletal complaints in this occupational group is high; in the UK, 74% of cleaners reported pain and discomfort in the last 12 months and 53% in the last 7 days [4]. In Taiwan, almost 90% of cleaners suffer from musculoskeletal complaints [9]; the majority of these associated these complaints with their profession [9]. Frequent complaint regions were the hand/wrist (41.7%), shoulder (41.1%), lower back (37.8%) and elbow (33.3%) [9]. Also, in the studies of Pekkarinen [10] and Woods and Buckle [4], the shoulders, neck, back and arms were found to be the frequent locations of musculoskeletal disorders in cleaners. Furthermore, in the study by Mondelli et al. [11], carpal tunnel syndrome was found in 48% of cleaners.

During cleaning work, such as vacuuming, the upper extremities are heavily stressed. Bell and Steele [12] considered the work-related risk to the musculoskeletal system to be high. In addition, frequently bent postures were observed [4,10,13]. Pataro and Fernandes [14] identified extreme rotational and flexion combination postures as risk factors for lower back pain in urban cleaners.

Large sections of the vacuuming movement are executed by repetitive pushing and pulling [12,13,15,16]. These cyclic repetitive movement sections represent a potential risk for the musculoskeletal system [6,12,17]. Furthermore, the monotonous repetitive work and the “moving continuously from place to place” are perceived as particularly physically demanding by cleaners [10].

The vacuuming of free surfaces, in particular, resembles a cyclical movement. Therefore, Cabeças [18] used a modified version of the Strain Index (SI), which was originally developed for cyclic, mono-task occupations, to assess vacuuming. There are physiological reference values for the cyclical movement of walking at which an ergonomic movement can be estimated [19]. A generalization of the vacuuming movement to a movement profile, however, requires that inter- and intra-subject differences are small compared to the general range of movement. If, however, the intra-individual movement patterns between different conditions (vacuum cleaner and floor) show clear differences in the joints, which make a significant contribution to the movement ergonomics, then the movement ergonomics can be influenced by different conditions.

Therefore, the aim of this study was to quantify the movement during habitual vacuuming (habitual vacuuming with domestic vacuum cleaners (handstick and cylinder vacuum cleaners)) and to characterize the movement profile with regard to its inter- and intra-subject variability.

## 2. Materials and Methods

### 2.1. Subjects

Thirty-one volunteers (21 f/10 m) aged between 18 and 65 years with an average age of 33.4 ± 10.7 years participated in the study. The subjects were, on average, 172.8 ± 9.4 cm tall and 66.9 ± 13.9 kg of weight. Thirty subject were right-handed and one left-handed. All subjects had experience with vacuum cleaning, however none of the subjects were professional vacuum cleaners.

Exclusion criteria for the study were current injuries (herniated discs, spinal column injuries), rheumatic diseases, severely restrictive spinal deformities (scoliosis) or stiffened spinal column joints (pathological or surgical) and genetic muscle diseases. Exclusion criteria were queried via a questioner prior to the study.

Informed consent was obtained from all subjects participating in the study. A positive ethics recommendation from the Department of Medicine of the Goethe University Frankfurt am Main (ethic-number: 335/18) was obtained.

### 2.2. Measurement System

The kinematic data were collected with the inertial motion capture 3D motion analysis system MVN BIOMECH Link from XSens (Enschede, The Netherlands). This is a personal system which provides position and angle information as well as acceleration and speed parameters of the human body by means of 17 motion sensors. Sensors were placed into a suit provided by the company. The position of the sensors was on the top of the feet (left and right), at the shank (left and right), thigh (left and right), pelvis, at the spine in the height of the Th8, on the head (wristband), on the acromion (left and right), upper arm (left and right), lower arm (left and right) and the dorsum of the hand (left and right, using a glove).

The sampling rate of the system is 240 Hz and the measuring error is specified by the manufacturer as ±1%.

### 2.3. Vacuum Cleaners

In general, vacuum cleaners have similar components such as the handle, canister and floor nozzle. However, there are different designs of vacuum cleaners, especially between handstick and cylinder vacuum cleaners. While the canister of a handstick vacuum cleaner is integrated into the style and pipe, the hose of a cylinder vacuum cleaner connects the canister standing on the floor with the handle and pipe. Furthermore, all vacuum cleaners differ in dimensions, weight and adjustment controls.

**Figure 1:**
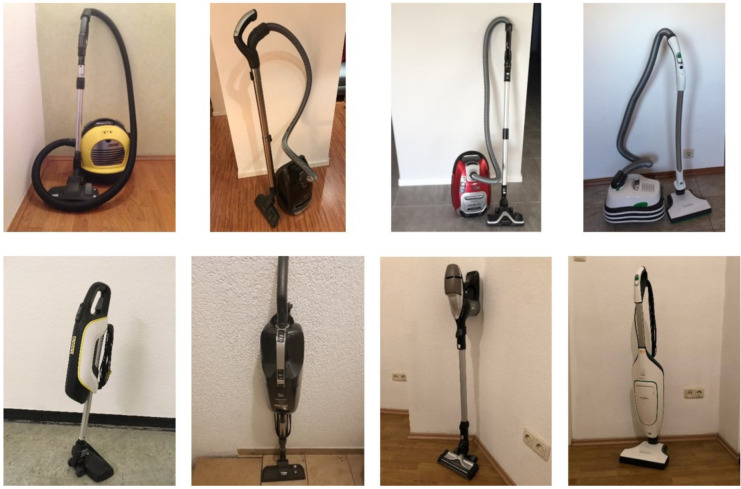
Eight different domestic vacuum cleaners (4 handstick and 4 cylinder vacuum cleaners) from four different manufacturers are shown in this figure.

To generate data, 8 different domestic vacuum cleaners (4 handstick and 4 cylinder vacuum cleaners) from four different manufacturers were used (Figure 1). Their properties are shown in Table 1.

### 2.4. Measuring Procedure

**Figure 2:**
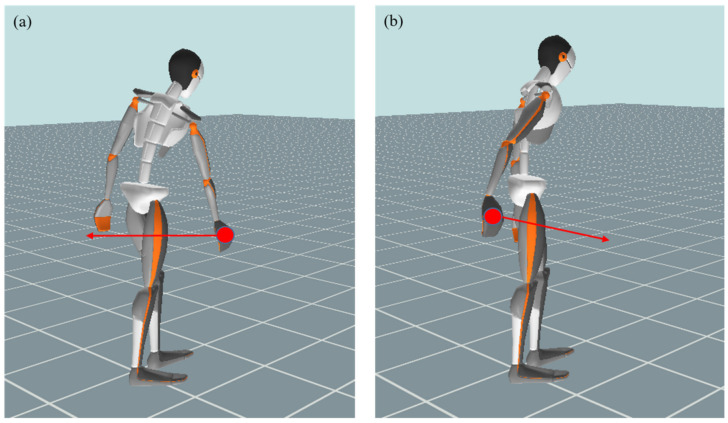
(**a**,**b**) Reference points and directions of movement in the habitual vacuuming cycle: (**a**) distal turning point and start of pulling phase, (**b**) proximal turning point and start of pushing phase.

Prior to measurements being taken, the subjects adjusted each vacuum cleaner handle to their preferred length. For standardized habitual vacuuming, the participants adopted a step position. The contralateral leg to the dominant hand was in front, taking care that both heels had ground contact. The foot position was not changed during the measurement. The participants performed the vacuuming movement in a habitual manner in the viewing direction (Figure 2). Twelve cycles with 8 vacuum cleaners (4 handstick and 4 cylinder vacuum cleaners each) of habitual vacuuming on a polyvinyl chloride (PVC) floor and on carpet were recorded. This resulted in a total of 16 different conditions (8 vacuum cleaners * 2 floors). Of the 12 recorded cycles, the mean 10 cycles were analysed (The first and the last cycle were removed. It was assumed, that the first and last movement are prominent to start and stop effects). The vacuum cleaners were used in a randomized order. The measurements were undertaken in a sequence, with a minute break in between different cleaners. Each participant had enough time to familiarize with each vacuum cleaner in advance of the data recordings. They could test vacuuming in advance for a few minutes. The participants were instructed to use their habitual movement amplitude (“vacuuming amplitude”) when vacuuming. They were not given a specific mark to follow, as the aim of the study is to establish the habitual vacuuming profile of the leading upper extremity side.

The participants were able to perform the vacuuming intuitively with one or both hands. The vacuuming movement was divided into individual movement cycles via the position of the hand marker in the direction of movement. The distal turning point was used as the start and end point, whilst the proximal inflection point was used to calculate the duration of the back and forth movement. The backward movement started at the distal turning point and ended at the proximal turning point, whilst the forward movement, correspondingly, started from the proximal and ended at the distal turning point.

### 2.5. Statistical Analysis

**Figure 3:**
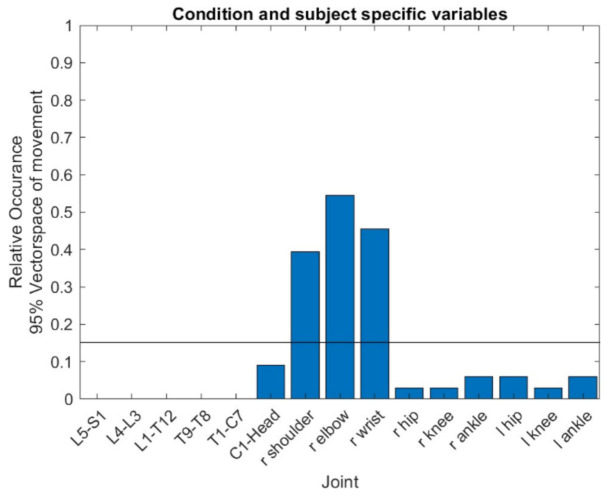
The relative frequencies of loadings in 15 joints beyond the limit of ± 0.045 of the Principal Component Analysis (PCA) to explain 95% of the motion variance (limit value of the relative frequencies at y = 5/33 = 0.1515).

Data preparation and statistical analysis were performed with MATLAB^®^ vR2018a software (The Mathworks Inc., Natick, MA, USA). Fifteen joints (Figure 3) and the vacuuming cycle distance and speed were analyzed. The vacuuming cycle distance was defined as the maximum elongation of the vacuuming cycle. The left upper extremity was not considered, as it was not relevant, especially for one-armed vacuuming. Each vacuuming cycle was time normalized to 100 time steps. In the case of the left-handed subject, the data of the left and right half of the body were mirrored. Cycles in which measured values deviated by more than 5 standard deviations of the entire data pool in the respective joint movement were defined as outliers and eliminated from the data set. If more than 3 cycles had outliers in a joint movement, the movement dimension in the respective record was completely removed.

For a general assessment of the inter- and intra- subject variability, the mean variabilities per condition, per subject and overall were calculated. The mean variability was calculated for every condition for each subject. Firstly, the standard deviation for every time point for every joint and dimension was calculated. The standard deviation was averaged over all time points. Following this, the average over all subjects, conditions and joints was calculated. For the intra-subject variability, the standard deviation was calculated across all conditions per subject and the average over all subjects, joints and dimensions was calculated. For the inter-subject variability, the standard deviation was calculated across all conditions and subjects and the average was built across joints and dimensions. Means and standard deviations were calculated for the vacuuming cycle distance and speed.

A Principal Component Analysis (PCA) was performed to detect the joints with the most variability between subjects and within subjects. The 100 time-normalized values * the 15 joint angles * the 3 dimensions used were displayed as a vector. The 10 repetitions * the 8 vacuum cleaners * the 2 conditions (floors) * the 31 subjects formed the trials. Thus, the input matrix was 4960 × 4500. Before the PCA, the mean value of all trials was subtracted from each input vector. The PCA, thus, characterizes the differences between the individual trials at each point in time. In the next step, the frequencies of the loadings were determined. A loading was deemed important when the eigenvector reached a value higher than three times the mean value of 0.045 (15 joints * 3 angular dimensions * 100 time steps = 4500 variables; limits of the charges at ± (3/√4500) = ± 0.045; Figure 2). Joints with a high motion variability were defined as those that exceeded the loading limits of ± 0.045 at least five times in the relevant PCA components (5/(number of eigenvectors that explain 95% of the variance)). These joints were represented in their movement dimensions by the mean movement (mean values over the 100 normalized points in time) and the range of standard deviation over the cycle.

The time normalized waveforms were statistically analysed by statistical parametric mapping (SPM) [20]. This technique allows the statistical interpretation of the whole waveform instead of the selection of a few discrete data points. The alpha value of significance was set to α = 0.05. The mean across the ten trials within each of the 8 cleaner conditions per subject was calculated. These 248 waveforms (31 subjects × 8 cleaner) were subjected to a paired student’s *t* test within the framework of the SPM. A student’s *t*-test was used to test between the duration of the forward and backward movement. The significance level was set to α = 0.05.

## 3. Results

The lengths of sections in the vacuuming cycle (forward and backward movement) averaged 0.65 ± 0.20 m. The maximum vacuuming speed was 1.28 ± 0.35 m per second for the forward movement and −1.29 ± 0.36 m per second for the backward movement. Accordingly, the duration of the forward motion was 49.5 ± 3% of the full cycle. The duration of the forward movement was not different to the duration of the backward movement t(495) = 0.46 (*p* = 0.64).

The mean variability per condition across all subjects, joints and dimensions was 1.5°. The intra-subject variability was 5.3° and the inter-subject variability was 5.9°. Therefore, the variability within a specific condition was small when compared to the intra- and inter-subject variability.

### 3.1. Principal Components of the Vacuuming Movement

The PCA determined that 95% of the variance in vacuuming motion could be explained by 33 components. Figure 2 shows the relative frequencies of the loadings above the defined limits of the 33 PCA components in the 15 joints. Three joints exceeded the limits far more than 5 times (or 0.15) in their loadings: the right shoulder (*n* = 13), the right elbow (*n* = 18) and the right wrist (*n* = 15). Therefore, these three joints are considered to vary significantly in the vacuuming movement (Figure 3).

### 3.2. Movements of the Right Upper Extremity in the Vacuuming Cycle

**Figure 4:**
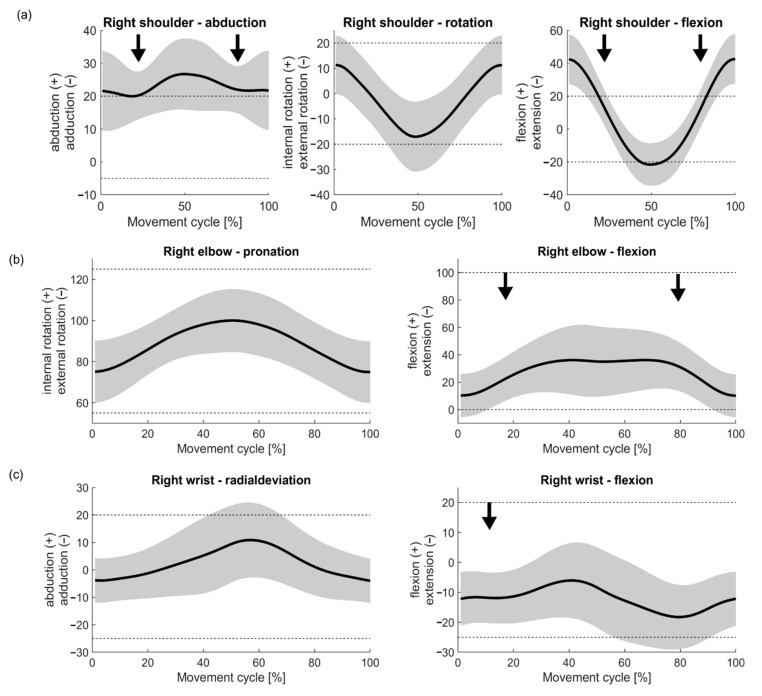
Movements of the right shoulder (**a**), elbow (**b**) and wrist (**c**) in the habitual vacuuming cycle (mean—solid line and standard deviation—grey area). The time dependent angles were time normalized to a full movement cycle. The dashed lines are the lower and upper limits for neutral movements. Movements within the neutral zone are considered ergonomically [21]. The black arrows point to time points of minimal standard deviation.

The joint movements of the right shoulder, elbow and wrist are shown in Figure 4a–c. Mean values and standard deviations at the cycle start/end (distal turning point) and at the mid-cycle (proximal turning point), as well as the mean standard deviation and its standard deviation over the whole cycle, are shown in Table 2.

The rotation of the right shoulder in the vacuuming cycle was relatively homogeneous. At the beginning and at the end of the cycle, the shoulder was rotated internally by an average of 11° ± 11° and, when the vacuum cleaner was returned to the body (centre of the cycle), the shoulder rotated externally by an average of 17° ± 13°. The scattering range was relatively consistent around the mean movement at an average distance of 11.7° ± 0.7° over the course of the cycle. During adduction and abduction, as well as flexion and extension in the shoulder, the scattering range became wider at the beginning of the cycle, in the middle of the cycle and at the end of the cycle but narrower in the transitions to the ends of the movement. At 0% and 50% of the motion cycle, the standard deviation of abduction was 12.4°. In the middle of the back and forth movement the standard deviation was 6.8° (black arrows in Figure 4a). The largest standard deviation of flexion and extension in the shoulder was at 14.3° at the most distant point and the smallest standard deviation at 9.7°, again, in the middle of the back and forth movement (black arrows in Figure 4a).

The elbow was in a pronation position during the whole vacuuming movement, which increased to the mid-cycle (proximal). The range of standard deviation was relatively consistent around the mean movement, on average 13.7° ± 1.0°. In elbow flexion and extension, the width of the deviation increased towards the mid-cycle (26.2° at 46% of the cycle), whilst it was relatively narrow at the distal positions (at the beginning and end of the cycle) (16.7° at 12% of the cycle) (black arrows in Figure 4b).

The wrist was found to move from an ulnar deviation (4° ± 8°) at the beginning of the cycle to a radial deviation (10° ± 14°) in the mid-cycle, with the width of standard deviation increasing towards the mid- cycle. The smallest standard deviation was 8.0° at 7% of the cycle and the largest at 14.2° at 45% of the cycle. The flexion and extension of the wrist was more in the form of a sine curve. The wrist was primarily in extension throughout the vacuuming cycle with a mean movement peak of 18.2° ± 11° at 80% of the cycle and a low point at 5.8° ± 13° (41% of the cycle). The standard deviation was wider in the middle of the cycle than at the beginning and end. The largest standard deviation of flexion and extension of the wrist was 13.7° at 56% of the cycle and the smallest at 8.4° at 11% of the cycle (black arrow in Figure 4c).

**Figure 5:**
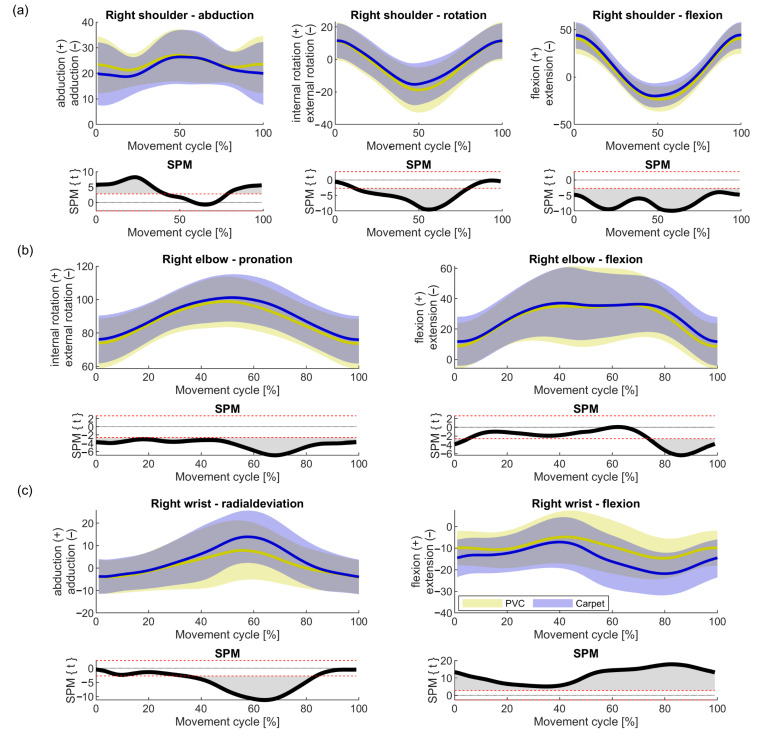
Movement profile of the two floor conditions for the right shoulder (**a**), elbow (**b**) and wrist (**c**) in the habitual vacuuming cycle (mean—solid line and standard deviation–area). The time dependent angles were time normalized to a full movement cycle. Blue indicates the carpet condition, yellow the polyvinyl chloride (PVC) condition. Positive values of the statistical parametric mapping (SPM) indicate greater angles for the PVC than for the carpet at the specific time point. Significant difference of clusters is reached when the z value of the SPM is outside the red dotted lines.

Significant differences could be detected between the two floor conditions (Figure 5, Table 3). In the right shoulder abduction two clusters could be detected. The PVC condition leads to an increase of the shoulder abduction in the pulling phase (1st half of the movement cycle) and in the end of the pushing phase (last 25% of the movement cycle) (Figure 5a, left). The shoulder rotation is reduced toward the proximal turning point (Figure 5a, middle). The PVC condition leads to a more extended flexion of the shoulder throughout the movement cycle (Figure 5a right). The elbow is more external rotated for the PVC condition (Figure 5b, left). The highest difference can be observed at the start of the forward movement. The elbow flexion shows two clusters (Figure 5b, right). However due to the cyclic movement, these two clusters are connected at the distal turning point (start of the movement cycle). In the PVC condition the elbow is more extended, especially at the end of the pushing phase. For the PVC condition the radial deviation of the wrist is smaller at the proximal turning point. Throughout the movement, the wrist flexion is higher for the PVC condition.

## 4. Discussion

The vacuuming distance and speed were relatively homogeneous in this study across all subjects and conditions. Thus, there were scarcely any differences in the back and forth movement of these parameters. The relative duration was approximately 50% in each case.

In the differentiated consideration of the variability of joints over all conditions, the movements of the lower extremities and especially of the back were quite constant. Therefore, the movements seem to be less influenced by these conditions, thus, the development of a general movement profile of these body regions is conceivable. In this study, the fixed foot position and the movement possibilities of the trunk when straight vacuuming in the plane, allowed less variability than under real conditions with its challenges to clean hard-to-reach areas. When vacuuming under real conditions, variations in movement and posture can be observed; bent, multi dimensionally twisted, unfavourable postures and movements have been described when vacuuming [8,10,13]. A movement profile of the lower extremities and back for more comfortable, standing vacuuming with a straight vacuuming direction, as performed in this study, can be useful in terms of an ergonomics evaluation. This profile could be used as a reference for vacuuming movements in real conditions that are likely to require more extreme body positions.

The two different floor conditions resulted in small but significant differences of the kinematics. Especially for the shoulder joint, the PVC condition increased the abduction angle slightly in the backward movement and increased the external rotation at the proximal turning point. Within the significant clusters of the SPM this led to a slightly less ergonomically cleaning condition. On the other hand, the reduction of the radial deviation at the proximal turning point and the increase of the flexion at the wrist in the forward movement shifted the movement towards the neutral position and therefore to a more ergonomical position.

In contrast to the lower extremities and the back, the upper extremity was highly variable. The joints of the active upper extremity show high inter- and intra-subject variability. Chang et al. [22] investigated the “natural product-use motions (NMs)” of four different vacuum cleaners, that is, the preferred product-use movement without using the actual product, compared to the “actual product-use motions (AMs)” using the product. The foot positions, five vacuuming directions and the vacuuming speed were specified. The intervals between the 5th and 95th percentiles of vacuuming motion were relatively wide for the upper extremity [22]. On the other hand, the intervals for the lower back, neck and especially the lower extremities were rather narrow [22]. This corresponds to the results of the present analysis in which the most variability in movement was found to be in the upper extremity. The generalization of a movement profile of vacuuming is therefore less meaningful for the upper extremity. It is noticeable that the variability of standard deviations is more inhomogeneous for movements of the upper extremity in flexion/extension and in abduction/adduction or radial-/ulnar deviation in the course of the vacuuming cycle. Rotation or pro-/supination movements are relatively homogeneous in the width of standard deviations over the cycle. Thus, a movement profile of the vacuuming cycle is probably conceivable for the latter parameters. The precision of the profile must be taken into account depending on the width of the deviation; even if the fluctuations of standard deviation over the entire cycle are relatively small, the mean standard deviation is 11.7° (rotation of the shoulder) or 13.7° (pro-/supination of the elbow).

Various publications show that the upper extremities are primarily exposed to household tasks such as vacuuming [5,7,9,12,18]. The high demands can have consequences for the musculoskeletal system. Thus, high prevalence of musculoskeletal complaints in the upper extremities can be observed among cleaners [4,9,10,13]. In this study, there was a high variability of motion of the upper extremity across all conditions and subjects. This suggests that movements of the upper extremity can be influenced by different conditions. A positive influence on movements can be important for the prevention of musculoskeletal complaints in cleaners. It is noticeable, especially at the turning points in the vacuuming cycle, that is, at the extrema of movements, that there were wider ranges of standard deviations than in the transitions. At the beginning and end of the cycle, as well as in the middle, broad scattering in the shoulder was observed in flexion/extension and abduction/adduction movements. At the mid-cycle, broad scattering was also observed in the wrist movements (flexion/extension, ulnar-/radial deviation) and in the flexion/extension of the elbow. Vacuum cleaner design, vacuum cleaner handling (single-hand vs. double-hand), vacuum cleaner (handle) adjustment, floor nozzle, floor characteristics, body proportions or individual movement patterns are all conceivable factors influencing movement. Since the intra- and inter-subject variabilities were substantially greater than the mean variability, it can be assumed that the conditions in particular, influenced the movements of the upper extremity. Product design is regarded as an important factor influencing posture, movement and physical strain during product use [12,16,22].

In comparison to the standard values of the range of motions of the upper extremities [23] the motion patterns of the upper extremity from this study are mostly not in the extreme areas of the range of motion. Only the elbow pronation is conspicuous which emerges clearly over the entire vacuuming cycle. The frequent repetitions of movements (taking into account the duration) due to cumulative strain and fatigue are associated with musculoskeletal discomfort and disorders during cleaning tasks [17], however the sole consideration of the extreme range of motions is not sufficient to identify physical hazardous body areas during vacuuming. Liu et al. [24] found an increased risk for carpal tunnel syndrome in computer workers who worked with a wrist extension of more than 20°. During the habitual vacuuming in this study, one direction of standard deviation of the wrist extension in the second half of the cycle was largely above 20°. However, the comparison between these two different tasks is difficult. While in computer workers, the task itself is rather static, the cleaning task itself is dynamic. Furthermore, the wrist angle extension reaches the critical value only sometimes. Therefore, no conclusions about the risk level in vacuum cleaning can be drawn.

In this study, kinematic data were the basis for examining a generalization of the movement profile of vacuuming with respect to ergonomic use. However, further factors have to be considered in the ergonomics of vacuum cleaning. In their systematic review of the interaction of force and repetition on the risks of musculoskeletal disorders, Gallagher and Heberger [6] pointed out that repetitive high-force tasks lead to musculoskeletal damage and disorder. Vacuum cleaner design can be crucial in force expenditure, thus, a vacuum cleaner with a high centre of mass requires higher muscle activity than one of a low centre of mass [16]. However, the force expenditure during vacuuming was not part of this study.

In future research, habitual vacuuming should be analysed in a more differentiated way, taking into account possible influencing factors. Chang et al. [22] developed a framework for usability evaluation concerning “natural product-use motions (NMs),” which could provide a basis for analysis. This framework considers product characteristics (design, user, environment, tasks), motion measurement and usability [22]. In addition to the physiological analysis of vacuuming, it is also useful to evaluate the subjective feeling of comfort in order to draw ergonomic conclusions.

This study was based on movements in a partial standardization (standardized foot position and vacuuming direction) of habitual vacuuming to generate comparable movement cycles. In order to examine a generalization of movement components during vacuuming, the less variable movements of the back and the lower extremities should also be considered in the future. In the overall context, it should be discussed whether a generally valid movement profile of vacuuming is only reasonable for certain body regions (e.g., the lower back) with less variability.

Only subjects with household cleaning experience were included but not professional cleaners. It can be speculated, that the number of hours using a vacuum cleaner does significantly influence the kinematics while cleaning. Therefore, further studies should include also a group of professional cleaners.

## 5. Conclusions

In congruence with the existing evidence, the present study confirms that the movements of the upper extremity in particular, vary during vacuuming. The variability of scattering of individual movement parameters of the upper extremity over the course of vacuuming cycle are partly homogeneous (rotational movements, distance, speed) and partly variable (flexion/extension, abduction/adduction). Due to the inconsistency of these results, a fundamental generalization of the movement profile during vacuuming does not appear to be meaningful. In the ergonomic evaluation of vacuuming, with the aim of preventing musculoskeletal complaints of the upper extremity, a multifactorial approach should be pursued which would also include environmental factors in addition to movement evaluation.

## Figures and Tables

**Table 1:** Properties of the vacuum cleaners according to the manufacturers’ specifications.

	Handstick Vacuum Cleaners				Cylinder Vacuum Cleaners			
	No.1	No.2	No.3	No.4	No.5	No.6	No.7	No.8
Length (cm)	18.2	55.4	85–109	24.0	49.6	38.3	43.0	29.8
Width (cm)	26.1	/	21	25.0	/	37.0	33.0	26.4
Height Min.—max. (cm)	62.1–123.4	16.5	15	125.0	22.7	37.3	26.0	52.8
Weight (kg)	3.2	5.02	3.0	2.9	7.26	6.6	6.0	7.9
Adjustable handle	yes	yes	yes	no	yes	yes	no	yes

**Table 2:** Mean values and standard deviations of movements of the right shoulder, elbow and wrist over the course of the cycle.

Joints	Dimensions	Mean ± SD at Cycle Start/End (Distal Turning Point) in Degree (°)	Mean ± SD at Mid-Cycle (Proximal Turning Point) in Degree (°)	Mean of SD ± SD (Over the Cycle) in Degree (°)
Right shoulder	Abduction (+)/adduction (−)	+21.4 ± 12.4	+27.4 ± 9.7	9.1 ± 1.6
	Internal rotation (+)/external rotation (−)	+11.2 ± 11.4	−17.4 ± 13.0	11.7 ± 0.7
	Flexion (+)/extension (−)	+42.4 ± 14.2	−22.4 ± 10.8	11.3 ± 1.3
Right elbow	Pronation (+)/supination (−)	+75.8 ± 15.1	+100.9 ± 14.4	13.7° ± 1.0
	Flexion (+)/extension (−)	+11.4 ± 17.1	+35.99 ± 25.6	20.2 ± 3.4
Right wrist	Radial deviation (+)/ulnar deviation (−)	−3.9° ± 8.2	10.0° ± 13.8	10.9 ± 2.2
	Flexion (+)/extension (−)	−12.3 ± 8.9	−8.6 ± 13.5	10.9 ± 1.8

**Table 3:** Results of the SPM applied to the two floor conditions. The SPM tests the difference between two conditions with respect to the smoothness. Therefore only statements about clusters can be made. The clusters mentioned in the table are labelled according to their occurrence from 0 to 100% in the movement cycle. The limits of significance are based on the waveforms of the individual joint directions. The *p*-value is only reported for significant clusters.

Joint and Direction	Area of SPM	Limit of Significant	*p*-Value	Degree of Freedom
shoulder abduction	Cluster 1	2.77	<0.001	[1 247]
	Cluster 2	2.77	0.008	[1 247]
shoulder rotation	Cluster 1	2.67	<0.001	[1 247]
shoulder flexion	Cluster 1	2.68	<0.001	[1 247]
elbow pronation	Cluster 1	2.64	<0.001	[1 247]
elbow flexion	Cluster 1	2.62	0.048	[1 247]
	Cluster 2	2.62	0.019	[1 247]
wrist radial deviation	Cluster 1	2.72	<0.001	[1 247]
wrist flexion	Cluster 1	2.73	<0.001	[1 247]
